# The influence of “momentum” on the game outcome while controlling for game types in basketball

**DOI:** 10.3389/fpsyg.2024.1412840

**Published:** 2024-06-14

**Authors:** Mingjia Qiu, Shaoliang Zhang, Qing Yi, Changjing Zhou, Mingxin Zhang

**Affiliations:** ^1^School of Athletic Performance, Shanghai University of Sport, Shanghai, China; ^2^Shanghai Key Lab of Human Performance, Shanghai University of Sport, Shanghai, China; ^3^Division of Sport Science and Physical Education, Tsinghua University, Beijing, China; ^4^College of Physical Education, Dalian University, Dalian, China

**Keywords:** momentum, sports performance analysis, winning factors, basketball, phenomenology

## Abstract

In competitive sports, momentum encompasses positive or negative changes in cognition, physiology, emotions, and behavior caused by sudden or a series of continuous events. Momentum occurring during basketball games leads to significant performance variation regarding positive net points differences for a specific team within a certain period. This study designed a quantitative framework based on two performative dimensions (time constraints and point differentials) to accurately identify momentum in basketball games, and explored the role of momentum in games. We identified 2,083 momentum occurrences in 372 professional elite basketball games. The number of momentum occurrences for winning teams is significantly higher than for losing teams (1.78 ± 0.47 Difference Value, *p* < 0.001); the correlation between momentum and game outcomes decreased as each quarter progressed. To distinguish the influence of contextual variables on momentum, we divided games into five types based on the team quality differences between the team and the opponent team. The decision tree model shows that first-quarter momentum is critical in games where weaker teams defeat stronger teams. This study provides insights for basketball coaches to formulate game strategies. More importantly, the momentum conceptual framework can help researchers identify and capture momentum, offering inspiration and reference for subsequent research.

## 1 Introduction

In competitive sports, the factors of winning have always been the focus of sports science. Previous studies have not only updated and enhanced our understanding of the fundamental rules of competitive sports from a theoretical perspective, but have also provided practical decision-making advice and training guidance for coaches and athletes. Basketball, a team sport involving on-court confrontation, is influenced by multiple factors affecting the game results (Brand et al., 2006; Gómez et al., 2008, 2011; Mikołajec et al., 2021). To comprehensively analyze the complex structure behind basketball game results, we need to consider the overall performance of both teams from both the offensive and defensive perspectives. Scoring and conceding points, as the most direct indicators of offensive and defensive effectiveness, are not only the final outcomes of all tactical performances but also the focus of competition between the two teams. However, scoring behavior is a dynamic, random, and non-linear complex process, with the frequency of scoring and the probability of consecutive scores being even more difficult to predict (Gabel and Redner, 2012; De Saá Guerra et al., 2013). Many researchers have attempted to understand the relationship between sports performance and game results by investigating the relationship between scoring and game context or by combining other performance indicators (Bar-Eli and Tractinsky, 2000; Sampaio et al., 2010b; Gómez et al., 2011, 2019).

Momentum is a scoring-related phenomenon in competitions; it is widely discussed by researchers with various definitions. Its manifestations in games include the “hot hand effect” (Gilovich et al., 1985; Koehler and Conley, 2003) and “winning streaks” (Salaga and Brown, 2018; Munoz et al., 2019). These different manifestations are based on various research subjects, focusing on different aspects, but they all share one common conclusion: momentum can alter individual and collective behavior. For example, the hot hand effect can influence players’ shot selection and coaches’ substitution strategies (Attali, 2013), and enhance athletes’ game performance (Zhao and Zhang, 2023). Further, scoring streaks can make athletes more positive, thereby improving their performance (Palmer et al., 2022). This evidence appears to prove one conclusion: in sports competition, success leads to further success. Momentum is a widespread phenomenon in sports competitions, but observed anomalous performances lack a unified concept or research framework. Even random processes like coin tossing can occasionally exhibit long winning streaks. Therefore, the anomalous performance of athletes or teams may also represent pure statistical probability (Bar-Eli et al., 2006; O’Donoghue and Brown, 2009).

The theoretical concept of momentum can be traced back to Adler’s serial conceptual model involving social action, where the sequential activation of motivation, emotion, and physiological arousal aims to enhance performance (Adler, 1981). Based on this theory, researchers in the field of sports psychology have internalized momentum as a psychological force that changes behavior and performance (Iso-Ahola and Blanchard, 1986; Taylor and Demick, 1994; Iso-Ahola and Dotson, 2016). Momentum is also viewed as a mediating phenomenon explaining the relationship between early success and subsequent success; it is a composite process and complex phenomenon involving psychological, physiological, and behavioral variables (Briki, 2017). Although momentum is the focus of several sociological and psychological studies, it also faces some skepticism, it is considered helpful for task completion but does not necessarily affect game outcomes (Gilovich et al., 1985; Koehler and Conley, 2003; Bar-Eli et al., 2006).

Whether in the field of sports performance or sports psychology, analyzing the role of momentum in achieving victory in sports competitions requires the ability to accurately capture momentum in games. Researchers have found that momentum is a concept that is difficult to quantify, and its ambiguous nature makes it challenging to accurately identify momentum in live games or game data (Moesch et al., 2014; Briki, 2017). Moreover, a controversy exists as to whether momentum is real or an illusion (Cotterill, 2012). Researchers have attempted to capture momentum through surveys and interviews with individual players and coaches, subjective feelings, and by observing game videos (Vallerand et al., 1988; Mace et al., 1992; Burke et al., 1997; Moesch and Apitzsch, 2012; Moesch et al., 2014). However, the fast-paced and ever-changing nature of basketball games makes it difficult to determine whether a team’s offensive or defensive performance has improved in short-term (Gómez et al., 2011). The statistical analysis of the sequence of game events is more objective and accurate (Mortimer and Burt, 2014; Csapo et al., 2015). Hence, this study used a quantitative formula constrained by time and score trends to accurately identify and capture momentum in basketball games. Then, we explored the relationship between momentum and basketball game outcomes and its role in different types of games. The formula used has been applied to study the short-term score difference variations between home and away teams (Chen and Fan, 2018). In this formula, $y\left(t\right)$ represents the point differential between the home team and away team at time t , s represents the increment of time, $y\left(s^{\prime }\right)$ represents the point differential between both teams at time $s^{\prime }$ , and μ represents the threshold value for momentum:


$$M\left(s^{\prime }\epsilon \left[t,s\right],\mu \right):=\frac{y\left(s^{\prime }\right)-y\left(t\right)}{s^{\prime }-t}\geq \mu$$

## 2 Methods

### 2.1 Research sample

The research sample consists of all regular season games of the 2021–2022 CBA season. After excluding playoff and overtime games, there are 372 valid games. The full game event data are obtained from public data provided by Beitai Tech to the CBA official website.^1^ This study used Python 3.0 (Python Software Foundation, version 3.0), a web scraping technology, to collect play-by-play event data for these 372 games and extract samples that met the criteria.

**Figure 1:**
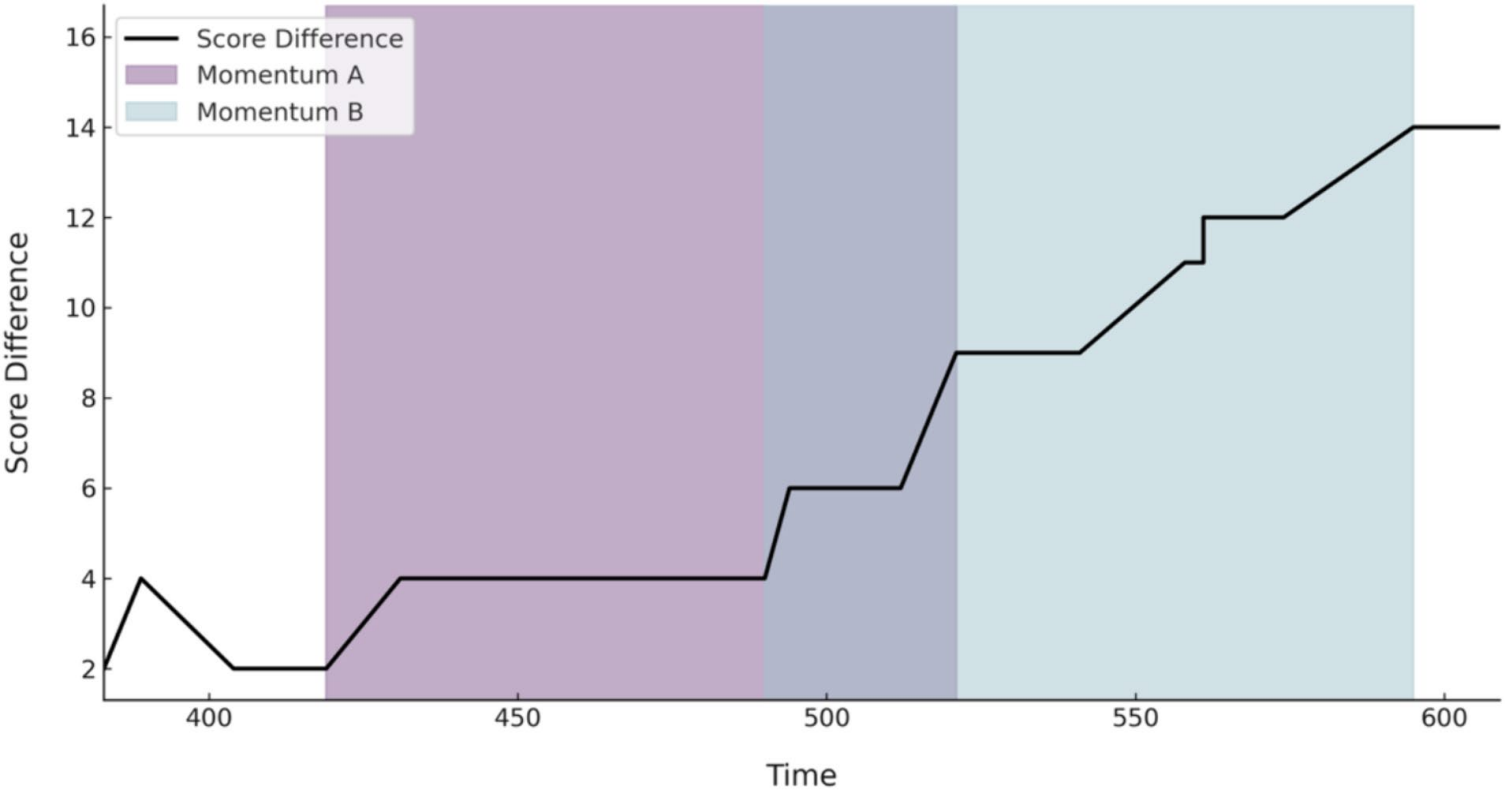
Cross momentum sketch map.

In the sports performance field, researchers have recognized the importance of three consecutive events for winning streaks and the hot hand effect (Carlson and Shu, 2007; Csapo et al., 2015). Therefore, this study posits that when setting the time constraints and score difference range for momentum, at least three complete offensive and defensive possessions should be included. In the 2021–2022 season of Chinese Basketball Association (CBA) games, the average duration of a complete offensive and defensive possession is about 32 s (data from CBA official website: https://www.cbaleague.com), and the average score per scoring possession is 2.1 points (data from Synergy: https://synergysports.com). Therefore, the minimum requirement for triggering momentum is achieving a net score difference of +6 points within 96 s. The net score is used because when discussing how momentum affects a game, one should not only consider a team’s score, but also the differences in offensive and defensive performance between both teams (Burke et al., 2003). The net score can intuitively reflect the results of the competition between both teams over time. Additionally, to explore the impact of momentum on game outcomes reasonably based on the researchers’ theory of “early success breeds later success,” we believe that when cross-momentum occurs (for individual team evaluations), the appearance of the first momentum leads to subsequent changes in competitive momentum (Adler, 1981; Richardson et al., 1988; Iso-Ahola and Dotson, 2014). For example, in Figure 1, there are two lines, “Momentum A” and “Momentum B” (Both from the same team), which fit the definition of momentum. We selected the first-initiated “Momentum A” as our sample and deleted “Momentum B.”

### 2.2 Reliability and validity of data

To determine the validity of the data from the CBA official website, four experienced basketball video analysts (all full-time video analysts for CBA teams) observed 20 randomly selected games, recording the game events and corresponding times for each game. Using the intraclass correlation coefficient to compare the recorded data with the CBA official website data, a high level of consistency was found (ICC = 0.97).

### 2.3 Statistical analysis

This study first conducted a descriptive analysis of the distribution of momentum in each quarter and the total number for the whole game, distinguishing between the winning and losing teams. Subsequently, a paired-sample Wilcoxon signed-rank test was used to determine if there were significant differences in the number of momentum occurrences between the two teams in each quarter. To observe the causes of momentum, the events of the first offensive and defensive possession when each momentum situation occurred were collected. A random forest was used to assess the importance of events to momentum strength. The significance level was set at *p* < 0.05.

To distinguish the game type, we first classified the 20 teams based on their winning rates (2021–2022 CBA regular season) using k-means clustering analysis (Sampaio et al., 2010a), with the elbow method was used to determine the optimal number of clusters. According to this analysis, team quality was automatically divided into three clusters, namely, the high winning rate teams as the Stronger Team, the medium winning rate teams as the Intermediate Team, and the low winning rate teams as the Weaker Team. Subsequently, the match between team quality and opponent team quality was used to determine the game type (Evenly Matched, Small Advantage/Disadvantage, Large Advantage/Disadvantage). In sports performance, distinguishing game type scenarios is a crucial step, as scenario variables can directly or indirectly affect the activities of teams and players (McGarry et al., 2013; O’Donoghue, 2014; Rein and Memmert, 2016; Gómez-Ruano, 2018). There are inherent differences in competitive strength due to differences in lineup strength and technical and tactical levels between the two teams in a basketball game. This difference is reflected not only in the game results but also in the game process. Hence, the game type scenario variable was controlled to distinguish the impact of these differences on the game.

The Kruskal–Wallis test was used to evaluate the differences in the number of momentum situations across five game types. Spearman’s correlation coefficient was used to analyze the correlation between momentum and game outcomes in each quarter; the thresholds for weak, moderate, strong, and very strong correlations were defined as 0–0.3, 0.3–0.5, 0.5–0.7, and ≥0.7, respectively. Decision tree models have been widely applied in sports performance analysis (Young et al., 2020; Matulaitis and Bietkis, 2021; Serna et al., 2021; Jetzke and Winter, 2022). In this study, the CHAID decision tree classification model helped distinguish the role of momentum in different game types and quarters. To run the CHAID decision tree classification model, the game outcomes were used as the dependent variable, and the number and its difference values of momentum were used as independent variables. The training and validation sets accounted for 80 and 20% of the sample, respectively. The maximum depth of the model was set to five levels, and the model was validated using cross-validation.

Statistical analyses were performed using Python (Python Software Foundation, version 3.0) and IBM SPSS software (version 25.0; SPSS Inc., Chicago, IL, United States).

## 3 Results

### 3.1 Data identification results

In the 2021–2022 CBA regular season, 2,083 valid momentum samples were obtained (no crossover of momentum event sequences between the two teams was found). Each sample contained a complete event sequence from the start event to the end event. Additionally, for the completeness of the study, the quarter and time of each event were extracted.

### 3.2 Statistical analysis results

Table 1 presents the descriptive statistics and paired-sample Wilcoxon signed-rank test results for the 2,083 samples from 372 games. The average number of momentum scenarios during the four quarters and overall momentum are both significantly higher for the winning team than for the losing team. From the first to the fourth quarter, the average momentum difference value between the winning and losing teams gradually decreased.

**Table 1:** Descriptive statistics and paired-sample Wilcoxon signed-rank test results.

	Sum		Mean ± *SD*		Difference value (W-L)		Paired-sample Wilcoxon Signed-Rank Test	
	*W*	*L*	*W*	*L*	Mean	*SD*	*p-*value	*Z*
Q1	360	160	0.97 ± 0.85	0.43 ± 0.58	0.54	0.27	<0.001	−8.605
Q2	351	170	0.95 ± 0.82	0.46 ± 0.65	0.49	0.17	<0.001	−7.973
Q3	349	176	0.94 ± 0.86	0.47 ± 0.65	0.47	0.21	<0.001	−7.302
Q4	313	204	0.84 ± 0.85	0.55 ± 0.69	0.29	0.16	<0.001	−4.624
Total	1,373	710	3.69 ± 1.65	1.91 ± 1.18	1.78	0.47	<0.001	−13.108

Table 2 presents the results of the random forest analysis. Free throw scouring has relatively high importance, followed by the opponent’s two-point shots made and the opponent’s turnovers.

**Table 2:** Random forest analysis results.

Feature	Importance
Free throw scoring	30.89%
Opponent’s two-point shots made	14.91%
Opponent’s turnovers	7.79%
Missed field goals	4.01%
Fouls on opponent’s shots	3.62%
Opponent’s three-point shots made	3.20%
Defensive rebounds	2.49%
Missed free throws	2.30%
Technical foul free throws	2.27%

Winning rate was used to determine team quality. Clustering analysis divided the 20 teams into three categories: Stronger Team (Cluster 2, *n* = 6, high winning rate = 75.0% ± 0.066), Medium Team (Cluster 1, *n* = 8, Intermediate winning rate = 54.9% ± 0.064), and Weaker Team (Cluster 0, *n* = 6, low winning rate = 18.4% ± 0.121). Furthermore, the match between a team’s quality and the opponent’s quality was used to determine different game types, namely (as shown in Table 3): Game Type 0 (Evenly Matched, *n* = 113), Game Type ±1 (Small advantage/Disadvantage, *n* = 187), and Game Type ±2 (Large advantage/Disadvantage, *n* = 72). For example, when the quality of the two competing teams is classified as Stronger Team and Weaker Team, the game type for the Stronger Team is +2 (large advantage), and for the Weaker Team is −2 (large disadvantage). This classification result groups similar game types together to explore the role of momentum in similar game situations.

**Table 3:** Classification of game type and situation based on quality of the competing teams.

Team quality (cluster)	Winning rate (Mean ± *SD*)	Opponent team quality	Game type	Game situation
Stronger team (cluster 2)	75.0% ± 0.066	Stronger team	0	Evenly matched
		Intermediate team	+1	Small advantage
		Weaker team	+2	Large advantage
Intermediate team (cluster 1)	54.9% ± 0.064	Stronger team	−1	Small disadvantage
		Intermediate team	0	Evenly matched
		Weaker team	+1	Small advantage
Weaker Team (cluster 0)	18.4% ± 0.121	Stronger team	−2	Large disadvantage
		Intermediate team	−1	Small disadvantage
		Weaker team	0	Evenly matched

**Figure 2:**
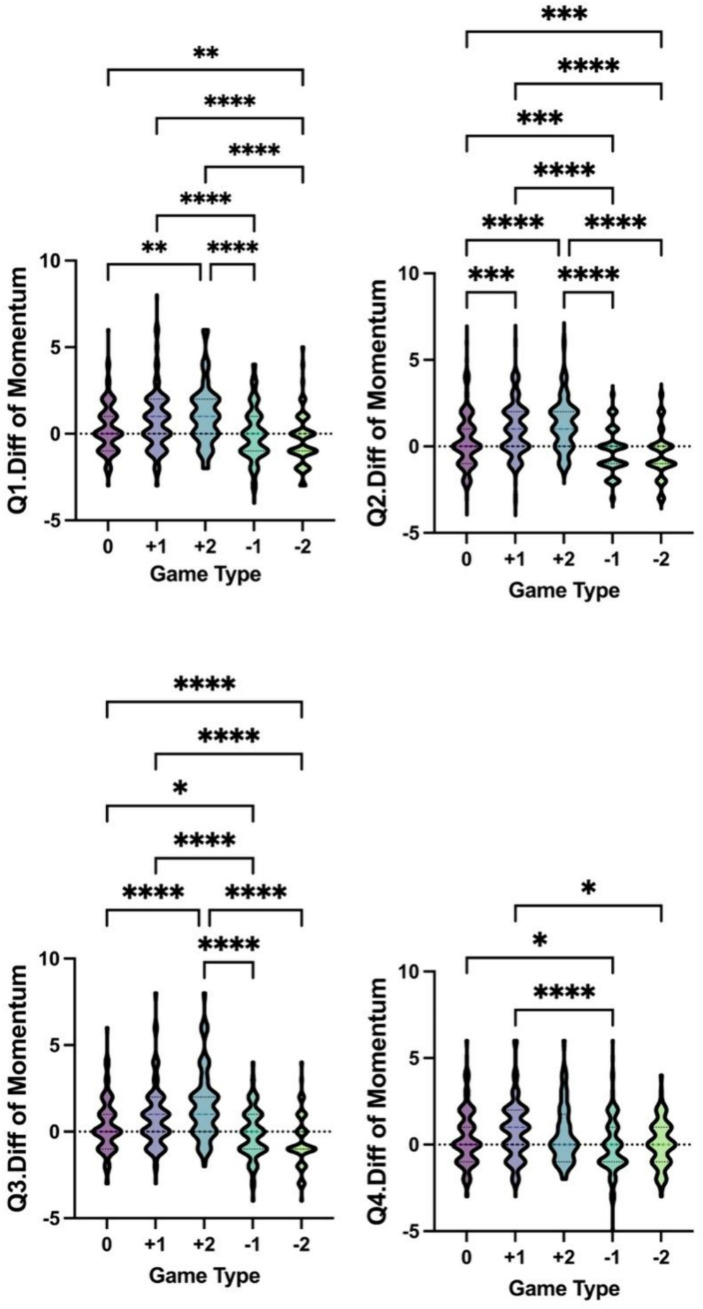
Kruskal–Wallis test results for differences in momentum among the five game types across the four quarters. In this figure, *p < 0.05, **p <0.01, ***p <0.001, ****p <0.0001.

The Kruskal–Wallis test was used to find the differences in momentum among the five game types across the four quarters. In the +1 and +2 game types, the differences in the number of momentum situations across the four quarters are consistently greater than 0. In other words, when facing weaker opponents, the likelihood of generating momentum is higher. Figure 2 shows the results of the Kruskal–Wallis test.

**Figure 3:**
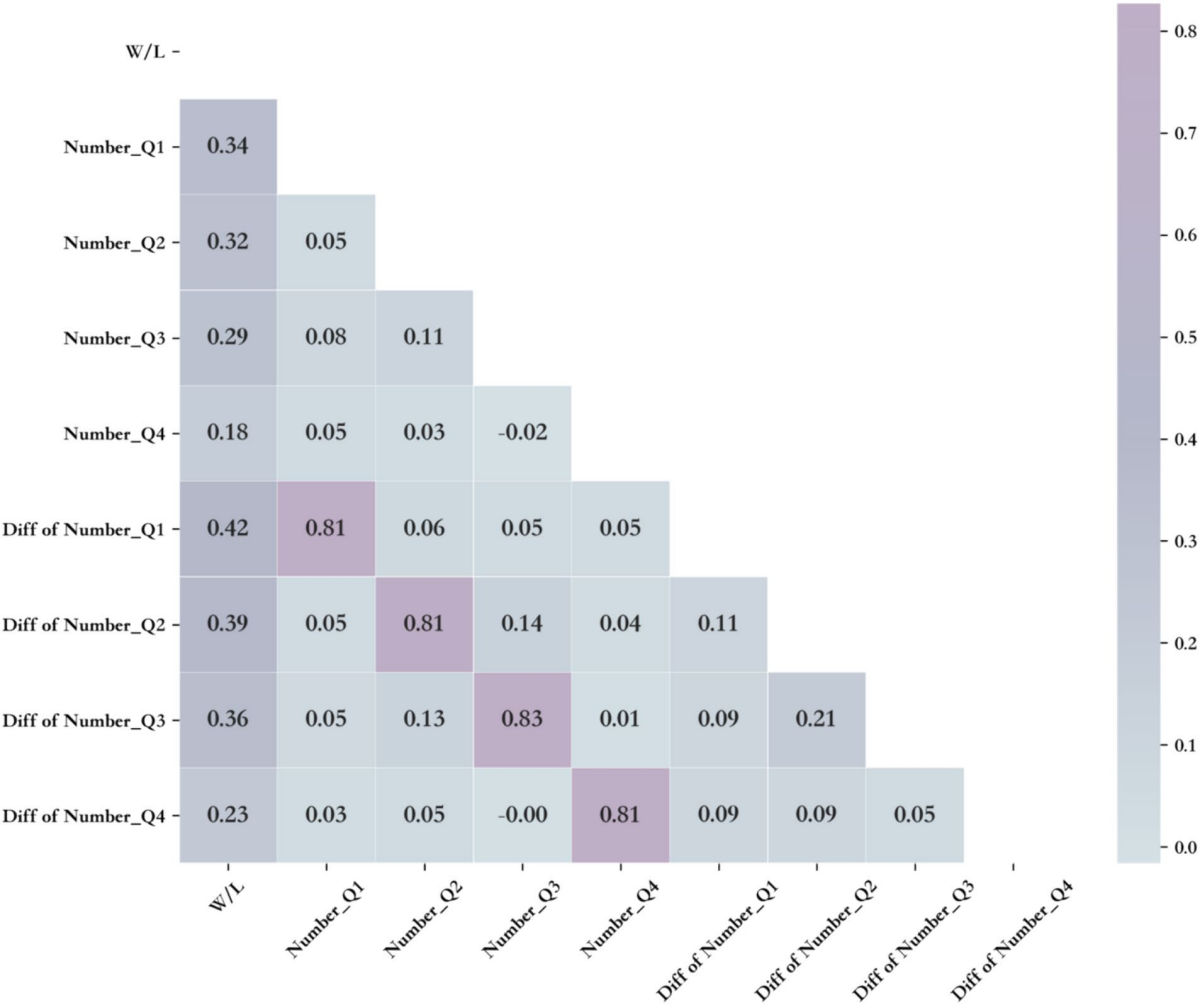
Spearman’s correlation analysis results.

The number of momentum situations and their difference values in the four quarters were used as feature variables. Spearman’s correlation coefficient was used to analyze the correlation between the features and the game outcomes. A correlation coefficient matrix was created and a heatmap was plotted (Figure 3). The correlation between the number and difference values of momentum in each quarter and the game outcome showed a decreasing trend, indicating that the momentum in the first quarter had the greatest impact on the game outcome.

**Figure 4:**
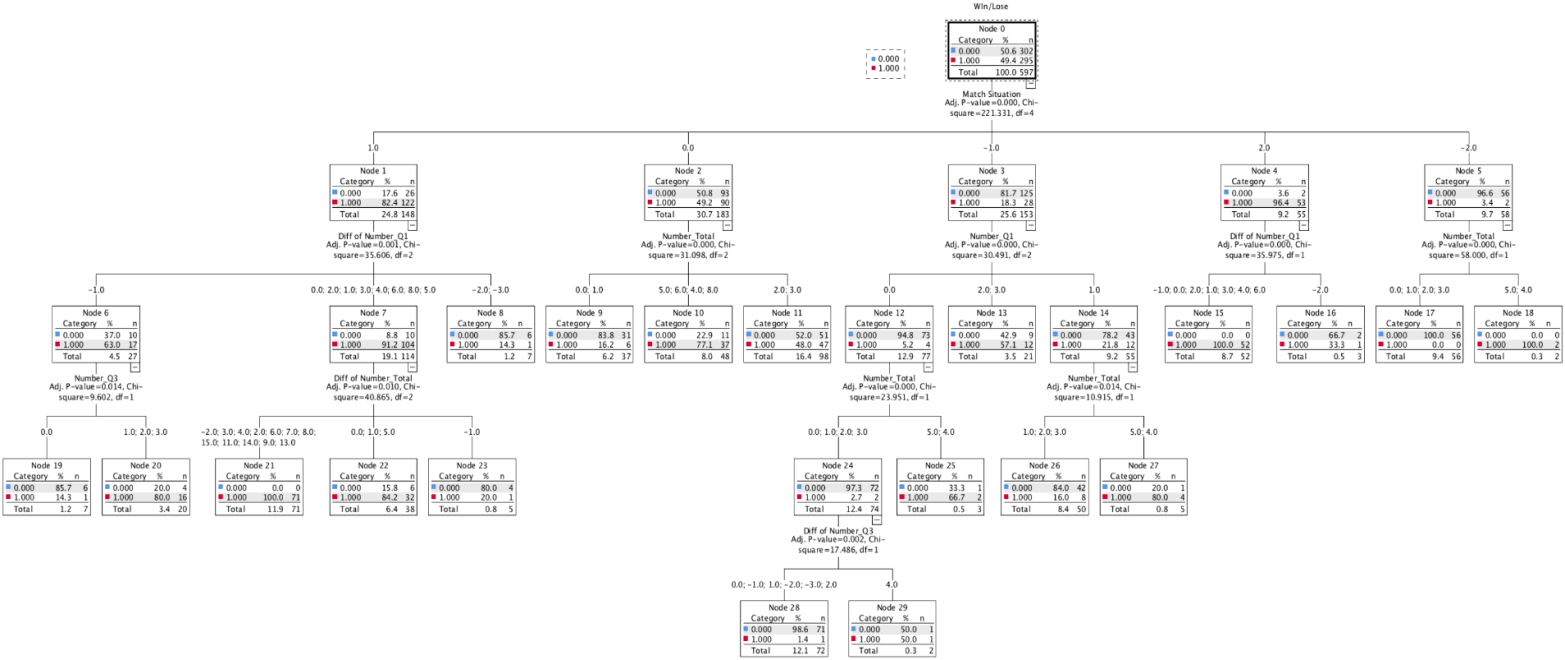
Results of the CHAID decision tree model with the outcome of the game as the independent variable.

Figure 4 shows the CHAID decision tree model. It uses game outcomes as the dependent variable, with the total sample divided into 80% training samples and 20% validation samples. The accuracy rates for the training and validation samples were 83.4 and 80.3%, respectively. The model contains 29 nodes, with the 5 game types as the root nodes. From the model results, it can be observed that in evenly matched situations, the total number of momentum situations can effectively aid in distinguishing between winning and losing samples. When the number of momentum situations is 4 or more, the win rate reaches 77.1%; when the number of momentum situations is 1 or 0, the win rate is only 16.2%. This indicates that striving for as many short-term scoring advantages as possible in the game positively impacts the game outcome. Additionally, there is no specific division of game quarters at this specific node level, indicating that in matches between equally matched teams, the number of momentum situations in different quarters has a minimal impact on the game. In contrast, when there is a certain quality gap between the two teams, momentum in the first quarter becomes an important factor in the “weaker beating stronger” scenario.

## 4 Discussion

Given the debate on momentum in previous research, we first aimed to present momentum in a quantifiable form to help establish an identifiable momentum conceptual framework based on relatively positive performance within specific time constraints and score change ranges. Then, using this conceptual framework, we accurately identified momentum in games and explored the role of this positive performance from the perspective of sports. The results of the difference test and correlation analysis showed that the momentum in the first quarter contributed the most to game outcome. After considering the impact of the game type scenario variables, the decision tree model further confirmed the importance of first-quarter momentum for weaker teams to defeat stronger teams. These findings provide a quantifiable method for identifying momentum, which aids sports performance scientists and psychologists in the further study of momentum, and helps coaches in formulating game strategies.

In matchups between evenly matched teams, offensive and defensive levels are relatively consistent, and the game is more likely to involve intense scoring battles, meaning frequent scoring exchanges between the two teams. This may be consistent with the changes in momentum between the two sides. In this situation, even if momentum is triggered several times (2–3 times), it may not affect the game outcome. Therefore, to gain an advantage in offensive and defensive levels and trigger more momentum, every detail in each quarter of the game becomes crucial, such as two-point shots and defensive rebounds (Lorenzo et al., 2010), assists (García et al., 2013), shooting percentage, and steals (Çene, 2018). In this context, momentum accumulation in a single quarter seems less important, and analyzing the impact of momentum on the game outcome by considering only one quarter is less effective than evaluating the overall value of momentum throughout the game.

The impact of momentum shows a changing trend at different stages of the game. Although the final stages of the game are considered very crucial (Bar-Eli and Tractinsky, 2000), especially the fourth quarter where every detail can affect the outcome (Ferreira et al., 2014), the influence of momentum on the game outcome decreases quarter by quarter. This observation has rarely been mentioned in previous studies. We acknowledge the unique characteristics of the first quarter of a basketball game because players, influenced by pre-game excitement, often exhibit higher motivation and better physical condition (Ben Abdelkrim et al., 2010), which usually reflects the true capabilities of the team (Martínez, 2014). The logic behind this unique characteristic may be related to the “success breeds success” theory reported by sports psychologists. Early success has been confirmed to influence the subsequent psychological state in sports competitions (Adler, 1981; Richardson et al., 1988; Iso-Ahola and Dotson, 2014; Morgulev, 2023). The importance of first-quarter momentum to the game outcome also supports this view from the perspective of sports performance analysis. The concept of momentum involves the psychological and emotional aspects of the game, reflecting the confidence and morale of the team and players, which are attributes difficult to quantify in the traditional definition of momentum.

Momentum has been a research topic in sports for over 40 years. It has always been considered a difficult concept to verify scientifically (Burke and Houseworth, 1995; Burke et al., 1997, 2003), but with the enrichment of statistical analysis methods and the exploration of contextual factors, researchers have been able to detect the existence of momentum through more objective and scientific empirical analysis (Hughes et al., 2006; Markman and Guenther, 2007; Dumangane et al., 2009; Morgulev et al., 2020; Gibbs et al., 2022; Weimer et al., 2023). Most of these empirical analyses are based on the dependence and non-stationarity of sequences. Sequence dependence refers to the idea that one event depends on adjacent events, while non-stationarity refers to the idea that the changes in success rate over the ongoing game exceed the possibility of being explained by chance (Moesch et al., 2014). Overall, both theoretical research and empirical analysis on momentum strongly emphasize the possibility that momentum may have a continuous structure (Iso-Ahola and Dotson, 2014, 2016). Clearly, sequences of micro-level events support the formation of momentum, and the value of these sequences lies in their representation of specific continuous events and behaviors in games, revealing the dynamic process of the game. Based on this viewpoint, establishing a momentum framework and then studying whether there are changes in the resulting sports performance, behavior, or psychology may aid in better understanding the momentum phenomenon, thereby making greater progress in theoretical research and practical applications.

The momentum framework in this study reflects the differences in offensive and defensive performance between two teams in a short period, testing the players’ continuous scoring ability and defensive quality. This is what differentiates the momentum framework defined in this study from the hot hand effect. Emphasizing the importance of defense in momentum is inspired by the study of Burke and Burke (1999) and Burke et al. (2003). We also attempted to use linear regression and Lasso models to determine the impact of defensive metrics on momentum. Although the model results showed that the opponent’s shooting scores had a significant negative impact on momentum and the opponent’s turnovers had a significant positive impact. However, the explanatory power of the models was low, so we could not rigorously confirm the specific impact of these metrics. This may be due to the sample size limitations or reflect the limitations of such models in studying momentum impact metrics. This aspect needs to be improved and refined in future research.

## 5 Conclusion

This study presents momentum in a quantifiable form, which aids in establishing an identifiable momentum conceptual framework. Momentum is specifically defined as the relatively positive performance of the team in offense and defense within a specific period. Through this framework, momentum can be defined more accurately in games, and its impact on game outcomes can be further explored. The results show that the momentum in the first quarter contributes the most to the game outcome, especially when weaker teams defeat stronger teams. It also emphasizes that the influence of momentum decreases quarter by quarter in different stages of the game.

Overall, this study provides a different perspective for the scientific validation of momentum. Momentum is not just a subjective concept; through quantification methods, its role in the game can be objectively assessed. This provides a reference for coaches in formulating game strategies and also offers new research methods on sports performance for scientists and psychologists. Future research can further explore the influence of different contextual factors on momentum.

## 6 Limitations and future research

It should be acknowledged that there are some limitations in this study. Firstly, we were unable to conduct an in-depth study of the event sequences within the momentum, which could help in understanding the causes and cessation of momentum. Secondly, we did not examine the impact of timeouts on momentum, which could be very helpful for formulating game strategies. Furthermore, we lack a deeper study of cross momentum, which could help identify key factors in sustaining momentum. Finally, due to the small sample size and insufficient dimensions, we were unable to apply linear regression or Lasso models in this study. These models could help explore the impact of game events on momentum-related dependent variables, such as the duration of momentum.

With a sufficient sample size, the events within the momentum can be included in the analysis to explore the role of different events in generating and stopping momentum. With a sufficient sample size, future research can focus on the complete event sequences within the momentum, analyzing the impact of single events or combinations of events on momentum, and can also compare events outside of momentum to further understand how to create momentum in a game. Furthermore, in-depth studies on the performance of momentum in different sports and how to apply this quantification method to other fields can be conducted.

## Data availability statement

The datasets presented in this article are not readily available because the data that support the findings of this study are available from the corresponding author, MZ, upon reasonable request. Requests to access the datasets should be directed to zhangmingxin@sus.edu.cn.

## Author contributions

MQ: Conceptualization, Investigation, Validation, Visualization, Writing – original draft. SZ: Methodology, Software, Writing – review & editing. QY: Data curation, Writing – review & editing. CZ: Formal analysis, Writing – review & editing. MZ: Conceptualization, Funding acquisition, Project administration, Resources, Writing – review & editing.

## Footnotes

^1^ https://www.cbaleague.com
